# Elucidating the interactive impact of tillage, residue retention and system intensification on pearl millet yield stability and biofortification under rainfed agro-ecosystems

**DOI:** 10.3389/fnut.2023.1205926

**Published:** 2023-08-21

**Authors:** Akshay K. Yogi, Ram Swaroop Bana, Samarth Godara, Seema Sangwan, Anil K. Choudhary, Ravi C. Nirmal, Shanti D. Bamboriya, Yashbir S. Shivay, Deepak Singh, Teekam Singh, Achchhelal Yadav, Shivani Nagar, Nirupma Singh

**Affiliations:** ^1^Division of Agronomy, Indian Council of Agricultural Research-Indian Agricultural Research Institute, New Delhi, India; ^2^Division of Computer Applications, Indian Council of Agricultural Research-Indian Agricultural Statistics Research Institute, New Delhi, India; ^3^Division of Microbiology, Indian Council of Agricultural Research-Indian Agricultural Research Institute, New Delhi, India; ^4^Division of Crop Production, Indian Council of Agricultural Research-Central Potato Research Institute, Shimla, Himachal Pradesh, India; ^5^Indian Council of Agricultural Research-Indian Maize Research Institute, Ludhiana, Punjab, India; ^6^Division of Agricultural Physics, Indian Council of Agricultural Research-Indian Agricultural Research Institute, New Delhi, India; ^7^Division of Plant Physiology, Indian Council of Agricultural Research-Indian Agricultural Research Institute, New Delhi, India; ^8^Division of Genetics, Indian Council of Agricultural Research-Indian Agricultural Research Institute, New Delhi, India

**Keywords:** conservation agriculture, nutrient biofortification, pearl millet, system intensification, residue retention, zero tillage

## Abstract

Micronutrient malnutrition and suboptimal yields pose significant challenges in rainfed cropping systems worldwide. To address these issues, the implementation of climate-smart management strategies such as conservation agriculture (CA) and system intensification of millet cropping systems is crucial. In this study, we investigated the effects of different system intensification options, residue management, and contrasting tillage practices on pearl millet yield stability, biofortification, and the fatty acid profile of the pearl millet. ZT systems with intercropping of legumes (cluster bean, cowpea, and chickpea) significantly increased productivity (7–12.5%), micronutrient biofortification [Fe (12.5%), Zn (4.9–12.2%), Mn (3.1–6.7%), and Cu (8.3–16.7%)], protein content (2.2–9.9%), oil content (1.3%), and fatty acid profile of pearl millet grains compared to conventional tillage (CT)-based systems with sole cropping. The interactive effect of tillage, residue retention, and system intensification analyzed using GGE statistical analysis revealed that the best combination for achieving stable yields and micronutrient fortification was residue retention in both (wet and dry) seasons coupled with a ZT pearl millet + cowpea–mustard (both with and without barley intercropping) system. In conclusion, ZT combined with residue recycling and legume intercropping can be recommended as an effective approach to achieve stable yield levels and enhance the biofortification of pearl millet in rainfed agroecosystems of South Asia.

## 1. Introduction

Malnutrition, lower productivity, and recurrent crop failures due to insufficient soil moisture are the predominant challenges in the water-deficit agroecologies across the globe. The twin catastrophes of climate change and the COVID-19 pandemic have further aggravated these problems in ecologically and economically fragile regions like South Asia and Africa (1). The restrictions imposed due to the pandemic have impeded food production, distribution, and trade, exacerbating the already dismal situation caused by climate-related catastrophic events. As weather patterns shift and ecological systems undergo physiological adaptations, the productivity of these regions has been severely impacted, leading to increased malnutrition and compromised food security (1, 2). Transformative changes in major food systems are necessary to build resilience and ensure equitable access to nutritious food for all (3). Micronutrient deficiencies, particularly zinc (Zn) and iron (Fe), rank among the leading causes of illness and disease in developing economies (4, 5). Globally, ~17.3% of the population suffers from zinc deficiency alone (6). Over 828 million people are estimated to be undernourished worldwide.^1^ To tackle these challenges, new fortification and system resilience approaches are needed in addition to cultivating nutrient-rich and high-yield-producing food crops (4). Micronutrient malnutrition remains a pressing issue due to low nutrient content and bioavailability in staple food grains, especially in arid and semi-arid regions where diverse and nutritious food options are limited (4, 5, 7–9). Biofortification of major food crops through climate-smart ecological approaches is a promising pathway to ensure affordable nutrition (4, 7, 8, 10, 11). Food fortification offers the advantage of delivering nutrients to large populations without drastic changes in food consumption patterns and minimal production costs (11, 12).

In rainfed regions of India, Africa, and Latin America, marginalized communities and small farmers heavily rely on millet and millet-based cropping systems for nutritional security (8, 13). Pearl millet [*Pennisetum glaucum* (L.) R. Br.], a major cereal crop of rainfed drylands, covers ~30 million hectares in Asia and Africa, with India accounting for around 30% of the total area (14). It exhibits inherent resilience to climate change and water stress and contains relatively higher levels of Zn, Fe, and proteins as compared to other cereals, such as wheat, rice, and maize (8, 9, 15). Additionally, it contains phenols and antioxidant compounds that are essential for human immune health (8). Among the leading production systems of semi-arid ecologies of the Indian sub-continent, the pearl millet-Indian mustard [*Brassica juncea* (L.) Czern & Coss] cropping system (PMCS) is predominant. This production system is practiced over a million-hectare area, mainly in tropical regions of India characterized by undulating light- to medium-textured soils, water scarcity, and poor soil fertility (16). This system faces challenges such as intermittent hydrothermal stresses, sub-optimal nutrient utilization, unstable productivity, and below-par quality of economic produce (14).

CA has been advocated as a solution to overcome these issues in rainfed dry-land regions (9, 13, 14). CA helps conserve soil moisture through crop residue retention (CRR), reducing evaporative water losses (9, 14), moderating thermal effects (14, 15), altering weed flora (17), and improving soil health (7). Despite the global advocacy for CA in diverse ecologies, research in India has primarily focused on irrigated agro-ecosystems, neglecting rainfed farming systems. There is a lack of systematic information on the various aspects of CA in the PMCS, including nutrient and moisture dynamics in the soil-plant system, yield stability, and crop quality. Diversification with legumes and cereals within CA and a system intensification approach could provide effective alternatives for climate resilience, higher productivity, and nutritious food (15, 18).

The association of legumes with their natural colonizing microorganisms appears to be a powerful combination for a sustainable and eco-friendly approach to cope with climate change effects on crops and improve plant nutrition (19). Legumes play a vital ecological role in improving the chemical and biological functions of the soil-plant-atmospheric continuum, in addition to their rich nutritional value (18). Crop management practices such as tillage, crop rotations, legume inclusion, and CRR enhance crop quality and nutrient assimilation in plant parts (20). The improvement in micronutrient content under ZT, in particular, has been associated with enhanced microbial activity and nutrient release during the decomposition process of crop residues (1, 21). Legume-imbedded systems fix more N with the addition of sufficient biomass with a narrow C:N ratio (22). This expedites the biomass decomposition with more C-sequestration and more micronutrient acquisition (23). The resultant soil organic matter (SOM) assists in the synthesis of organic acids in the rhizosphere, which in turn behave as micronutrient chelates, influencing the translocation and remobilization of micronutrients. It is hypothesized that the interaction between tillage, residue retention, and system intensification can sustain higher crop productivity through moisture conservation, less weed infestation, higher water use efficiency (WUE), and greater nutrient recycling while augmenting the micronutrient and protein content in the edible portion of the crops. The independent effects of these three factors are known to positively impact soil moisture conservation, weed infestation, and nutrient acquisition by plants. Additionally, this study focuses on providing integrative solutions for climate change and food security by utilizing the innovative approaches of sustainable intensification, tillage configuration, and cropping system-based diversification to achieve food biofortification and yield stability in prevalent millet systems. We also hypothesized that intercropping under ZT and CRR could enhance crop yield, micronutrient uptake, and grain quality. These practices are expected to enhance soil structure, increase soil organic matter content, and subsequently lead to significant improvements in crop yield stabilization, biofortification, and grain quality.

This study aims to investigate the effects of contrasting tillage systems, residue management options, and system intensification on pearl millet fatty acid and micronutrient contents, yield stability, and grain quality. By exploring diverse intensification alternatives, the research will provide a theoretical basis for selecting suitable cropping systems and tillage practices in rainfed areas. Furthermore, the study examines the impact of crop residue retention on yield stability, grain quality, fatty acid contents, and micronutrient profiles of pearl millet within the pearl millet-mustard production system in a cropping system mode.

## 2. Materials and methods

### 2.1. Experimental site and climatic conditions

**Figure 1:**
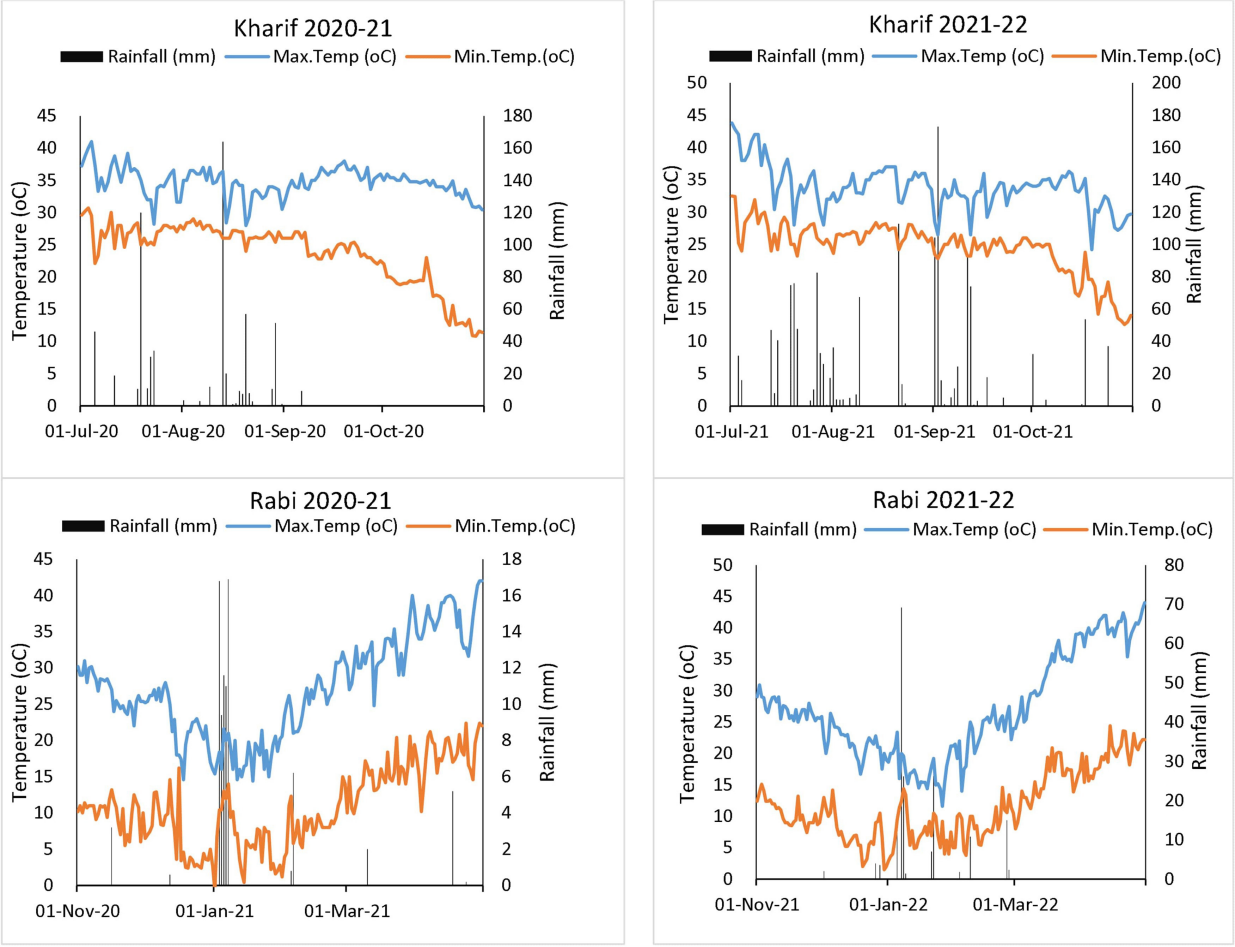
Mean monthly weather parameters recorded during the crop seasons of experimental years 2020–2021 and 2021–2022.

A 2-year field experiment (2020–2021 and 2021–2022) was conducted at the ICAR-Indian Agricultural Research Institute (ICAR-IARI), New Delhi (28°35′ N latitude, 77°12′ E longitude, 229 m altitude). The experimental site is located in a sub-tropical semi-arid climate characterized by hot and dry summer and cold winter seasons, with a mean annual precipitation of ~652 mm. The soil of the experimental site is sandy-loam in texture and taxonomically classified as a Typic Haplustept of Gangetic alluvial origin (24). The description of the initial soil characteristics of the experimental field is given in Table 1, while the weather conditions (rainfall, minimum, and maximum temperatures) of the *Kharif* and *Rabi* seasons are presented in Figure 1.

**Table 1:** Initial soil properties of the experiment field.

**Soil properties**		**CT**		**ZT**	**Method**
**Soil chemical properties**					
Available N (kg ha^−1^)		164.8		212.7	Alkaline KMnO_4_ (25)
Available P (kg ha^−1^)		14.9		15.1	Bray's No. 1 method (26)
Available K (kg ha^−1^)		172.4		183.2	Neutral NH_4_OAc (27)
Available S (kg ha^−1^)		17.6		19.2	(28)
Soil pH		7.2		6.7	(29)
Soil EC		0.16		0.24	(30)
Available micronutrients (mg kg^−1^) soil	Fe	4.32	Fe	4.92	DTPA extraction (31)
	Zn	0.47	Zn	0.65	
	Mn	4.12	Mn	5.16	
	Cu	1.14	Cu	1.36	
**Soil biological properties**					
SMBC (μg g^−1^ soil)		176.8		194.8	(32)
Dehydrogenase (μg TPF g^−1^ soil day^−1^)		26.2		35.4	(33)
Alkaline phosphatase (μg p-NPP g^−1^ soil h^−1^)		67.3		84.7	(34)

### 2.2. Crop and soil management

As rainfed crops, pearl millet, cluster bean, and cowpea were sown during the *Kharif* (July–October) season. Winter season crops (Indian mustard, chickpea, and barley) were also grown as rainfed crops; however, a pre-sowing irrigation of 50 mm depth was applied to ensure uniform germination and crop establishment. The triplicate experiment was laid out in a split-plot design with cropping systems and tillage configurations as the main plot (six treatments) and crop residue management as the sub plot treatments (three). Field layout details are explained in Supplementary Figure 1. CA practice encompasses not tilling the soil, rotating crops over the years, and leaving crop residues on the surface (35). In ZT plots, no preparatory tillage operations were carried out. CT is totally different from ZT practice. In CT plots, one deep plowing, two passes of harrowing, and planking were performed to have a uniform seedbed of fine tilth, and crop residues were incorporated by the rotavator. Mustard crop residues (2 Mg ha^−1^) from the previous crop were incorporated into CT and retained in ZT plots. For each sub plot (48 m^2^), 9.6 kg of crop residues (required rate of 2 Mg ha^−1^) were weighed, chopped, and applied. The pearl millet cultivar “*Pusa 443*” and the mustard cultivar “*Pusa mustard 28”* were sown with a seed rate of 4 kg ha^−1^ for each crop. A detailed description of the treatments is given in Table 2. The timeline (sowing and harvesting) is shown in Supplementary Figure 2. The row-to-row and plant-to-plant spacing of 50 and 30 cm, respectively, was adopted. The sowing was performed using a seed-cum-fertilizer drill in CT plots and a 9-tyne zero-till planter in ZT plots. The fertilizer application was made based on the soil test values of the experimental plots. The pearl millet and mustard crops received fertilizers at the rates of 60:40:40 and 80:40:40 kg ha^−1^ of N:P:K, respectively. The sources of nutrients were urea (46% N), single superphosphate (SSP, 16% P_2_O_5_), and muriate of potash (MOP, 60% K_2_O). In the *Kharif* season, two-thirds of the nitrogen (N) and total doses of P_2_O_5_ and K_2_O were applied as basal. The remaining one-third N-dose was applied 5–6 weeks after sowing, based on the soil moisture status of the experiment field. The crop residue nutrient contents (N, P, and K) have been provided in Supplementary Table 1.

**Table 2:** Treatment details of the experiment.

**No**.	**Treatment**	**Description**		**Short-term**
**Cropping system and tillage configuration (main plot)**				
1	Conventional tillage (CT)-based pearl millet-mustard cropping system	Crop residues were applied and incorporated through one deep plowing, two harrowings, and planking.		CT (P – M)
2	Zero tillage (ZT)-based pearl millet—mustard	No plowing during both years; zero-till planter sowing, pearl millet and mustard as monocrops.		ZT (P – M)
3	ZT-based (pearl millet + cowpea – mustard + barley)	No plowing during both years; zero-till planter sowing. Pearl millet + cowpea – [1:1] and mustard + barley – [4:3 row ratio]		ZT (P + C – M + B)
4	ZT (pearl millet – mustard + chickpea)	No plowing; zero-till planter sowing [pearl millet (100% plant population)] and mustard + chickpea [1:1 row ratio]		ZT (P – M + CP)
5	ZT (pearl millet + cluster bean – mustard)	No plowing, zero-till planter sowing. Pearl millet: cluster bean [1:1 row ratio]		ZT (P + CL – M)
6	ZT (pearl millet + cowpea – mustard)	No plowing, zero-till planter sowing [pearl millet: cowpea – 1:1 row ratio]		ZT (P + C – M)
**Residue intensity (sub plot)**				
1	*Kharif* season crop residue-retention	Mustard crop residue was applied and incorporated with conventional tillage treatment and surface retention in zero tillage treatment	Mustard crop residue-retention (2.26 Mg ha^−1^)	KR
2	*Rabi* season crop residue-retention	Pearl millet residues were applied and incorporated with conventional tillage treatments, and the surface was retained in zero tillage treatments	Pearl millet crop residue (2.0 Mg ha^−1^)	RR
3	Both season crop residue-retention	Crop residues of both pearl millet and mustard crops were used under the treatments CT (incorporated) and ZT (surface residue retention)	Mustard residue (*Kharif* season) 2.0 Mg ha^−1^ and pearl millet residue (*Rabi* season) 2.0 Mg ha^−1^)	BR
**Interaction treatment symbols (GGE analysis)**							
Treatment (main plot)	CT (P – M)	ZT (P – M)	ZT (P + C – M + B)	ZT (P – M + C)	ZT (P + CL – M)	ZT (P + C – M)
Sub plot	KR	1	2	3	4	5	6
	RR	7	8	9	10	11	12
	BR	13	14	15	16	17	18

### 2.3. Crop harvesting and yield estimation

Each year, the pearl millet, cowpea, and cluster bean crops were harvested manually at physiological maturity. The final grain yield was calculated by taking the grain moisture content at 12%. To compare different treatments under intercropping systems, the crop yields were converted to pearl millet equivalent yield (PEY) (36). Minimum support prices (MSP) for pearl millet, cowpea, and cluster bean as fixed by the government of India (data provided in Supplementary Table 2) were used to convert these crops' yields to PEY and summed up for system PEY. MSP is the minimum price set by the government for certain agricultural products, at which the products would be bought directly from the farmers if the open market prices were less than the cost incurred. Eq. 1 was used to estimate PEY as follows:


(1) $$\begin{array}{l} {\text{PEY (Mg ha}}^{-1}\text{) = Pearl millet yield}\\ \text{ +[(CY×Cp) +(CLY×CLp)]/Pp} \end{array}$$

where CY is the yield of cowpea (Mg ha^−1^), CLY is the yield of cluster bean (Mg ha^−1^), Cp is the cowpea MSP (INR kg^−1^), CLp is the cluster bean MSP (INR kg^−1^), and Pp is the MSP of pearl millet (INR kg^−1^).

### 2.4. Plant chemical analysis

To estimate N-concentration and crude protein content in plant parts, the plant samples were air-dried and oven-dried at 60 ± 2°C. Representative plant samples (0.5 g each) were digested with 10 ml of analytical-grade concentrated sulfuric acid combined with a digestion mixture (CuSO_4_ + K_2_SO_4_ + Selenium powder + Mercury oxide) and analyzed using Kjeldahl's apparatus as per the procedure described by Rana et al. (37). For micronutrient analysis in pearl millet grains and stover, the samples were taken at the crop harvest stage. The 0.5 g of finely ground (1 mm sieve) plant samples were taken and digested with the solution of concentrated HNO_3_ and HClO_4_ acids (in a 9:4 v/v ratio) in conical flasks. The flasks were kept on a digestion plate for heating up to 3.5 h, or until a colorless residue was left in the digestion vessel. After being cooled, the remaining substance was combined with a 0.1 N solution of H_2_SO_4_ and diluted to a final volume of 100 ml. The digested samples were taken to estimate micronutrients (Fe, Zn, Mn, and Cu) using atomic absorption spectrometry (AAS PLUS, Motras Scientific, India) as per the procedure described by Rana et al. (37). The resultant micronutrient content of grain and straw was converted to micronutrient uptake (g ha^−1^) using yield data from this study using Equations (2) and (3) as follows:


(2) $$\begin{array}{l} {\text{Micronutrient content (mg to g kg}}^{\text{-1}}\text{)}\\ \text{= ppm × 0.001[\textbackslash{}1g = 1,000 ppm]} \end{array}$$

(3) $$\begin{array}{l} \text{Micronutrient uptake }(\text{g }{\text{ha}}^{\text{−1}}\text{= micronutrient}\\ \text{content }(\text{g }{\text{kg}}^{\text{−1}})\;\times \text{ yield }(\text{kg }{\text{ha}}^{\text{−1}}) \end{array}$$

For quality control, blank and replicated samples were run with each batch of samples and calibrated with standard solutions (0.2, 0.4, 0.6, 0.8, and 1.0, 5, 10 mg L^−1^ of mineral nutrients). The resultant precision was verified by analyzing three replicated samples. Additionally, glassware and flasks were thoroughly washed using strong oxidizing agents to prevent contamination. Certified standards, such as the 1,000 mg L^−1^ concentration of Zn, Fe, Mn, and Cu Certipur^®^ standard solution from Merck KGaA, EMD Millipore Corporation, Germany, were used for this purpose. The non-destructive method of oil estimation in whole seeds was carried out using a NIR transmittance grain analyzer (FOSS InfratecTM 1241) operating in the near-infrared region. This instrument was employed to determine the oil content of the seed samples.

### 2.5. Soil sampling and analysis

Soil samples were collected on July 2020 randomly from a well-established ZT field by taking five cores at 0–15 cm depth using a 5 cm diameter core sampler. The collected soil cores were mixed thoroughly, sieved (<2 mm), and divided into three sub-samples. One of the sub-samples was stored at 4°C before analysis of alkaline phosphatase activity, while another sub-sample was air-dried and analyzed for pH (37), organic carbon (37), oxidizable N (38), microbial biomass C (32), available P (39), K (27), and micronutrient content (32). The third sub-sample was used to determine the bulk density and soil moisture. Bulk density determination was done by the core method (37). Soil pH (water) was measured using a pH 700 Bench Meter (Eutech Instruments) at a soil:water ratio of 1:2.5. Soil samples were oven-dried and analyzed for nutrient contents as per the method described by Rana et al. (37). Total N was measured using the Kjeldahl method (38). Microbial biomass C was determined by chloroform fumigation and extraction (40). Soil total P and plant-available P content were determined by perchloric acid (HClO_4_) digestion (39) and the 0.5 M NaHCO_3_ extraction method (26), respectively, using a spectrophotometer. Dehydrogenase activity (DHA) was estimated by releasing triphenyl formazan and reducing 2,3,5-triphenyl tetrazolium chloride (33). Alkaline phosphatase activity was determined as described by Tabatabai (34).

### 2.6. Fatty acid profiling

For the fatty acids profiling of pearl millet grains, samples were esterified individually with methanol in the presence of concentrated sulfuric acid. Fatty acid esters were extracted with hexane from the reaction mixture and concentrated using a rotary evaporator (Heidolph, Germany). The fatty acid profile of the samples was analyzed in gas chromatography-mass spectrometry (GC-MS) using an 8010C GC (Agilent Technologies, USA) equipped with an HP-5MS column (60 m × 0.25 mm;/0.25 mm, Agilent Co., United States), which was directly connected to a triple-axis HED-EM 5975C mass spectrometer (Agilent Co., United States). The injection volume was 1 μl with flow mode in split control at 1:20. The carrier gas flow was set at 1.00 ml/min helium. Helium (high purity of >99.99%) was used as a carrier gas at a head pressure of 10 psi. The oven temperature was initially held at 80°C, and then increased with a ramping rate of 5°C/min until it reached 150°C and was held for 1 min. Again, the temperature was elevated with a gradient of 7°C/min to get 220°C. Finally, the temperature was raised to 320°C with an increment of 10°C/min. The MS acquisition parameters were as follows: ion source (150°C), electron ionization (70 eV), full scan mode (50–550 mass units), transfer line temperature (220°C), and EM voltage (1,250 V). Fatty acid esters were identified by matching their respective mass spectra from the NIST (National Institute of Standards and Technologies) mass spectral library (41).

### 2.7. Statistical analysis

Tukey's HSD test was used to identify variations in nutrient content (NC), uptake, and yield traits. The normality of the response variables was tested by the Bar graph method, and all the variables were found to be normally distributed. The significance and interactions between treatments were evaluated using a two-way ANOVA in a split plot design. All the effects were fixed effects used in the model. Differences between treatment means were compared using Tukey's HSD at a 5% probability level (*p* = 0.05). The SAS 9.3 statistical software package was used to analyze the data. R Studio version 2022.12.0 was used for the analysis of multivariate stability statistics (GGE biplot) (28, 42). GGE biplot analysis was computed using the “GGE Biplot GUI” package (28), with support from the helper application “RStudio” in the R statistical software. GGE biplot analysis was used to visually assess the presence of genotype × environment interaction, rank genotypes based on stability and mean in each treatment, and identify optimally performing combinations (43–45).

## 3. Results

### 3.1. Effects of tillage, CRR, and system intensification on crop productivity

**Figure 2:**
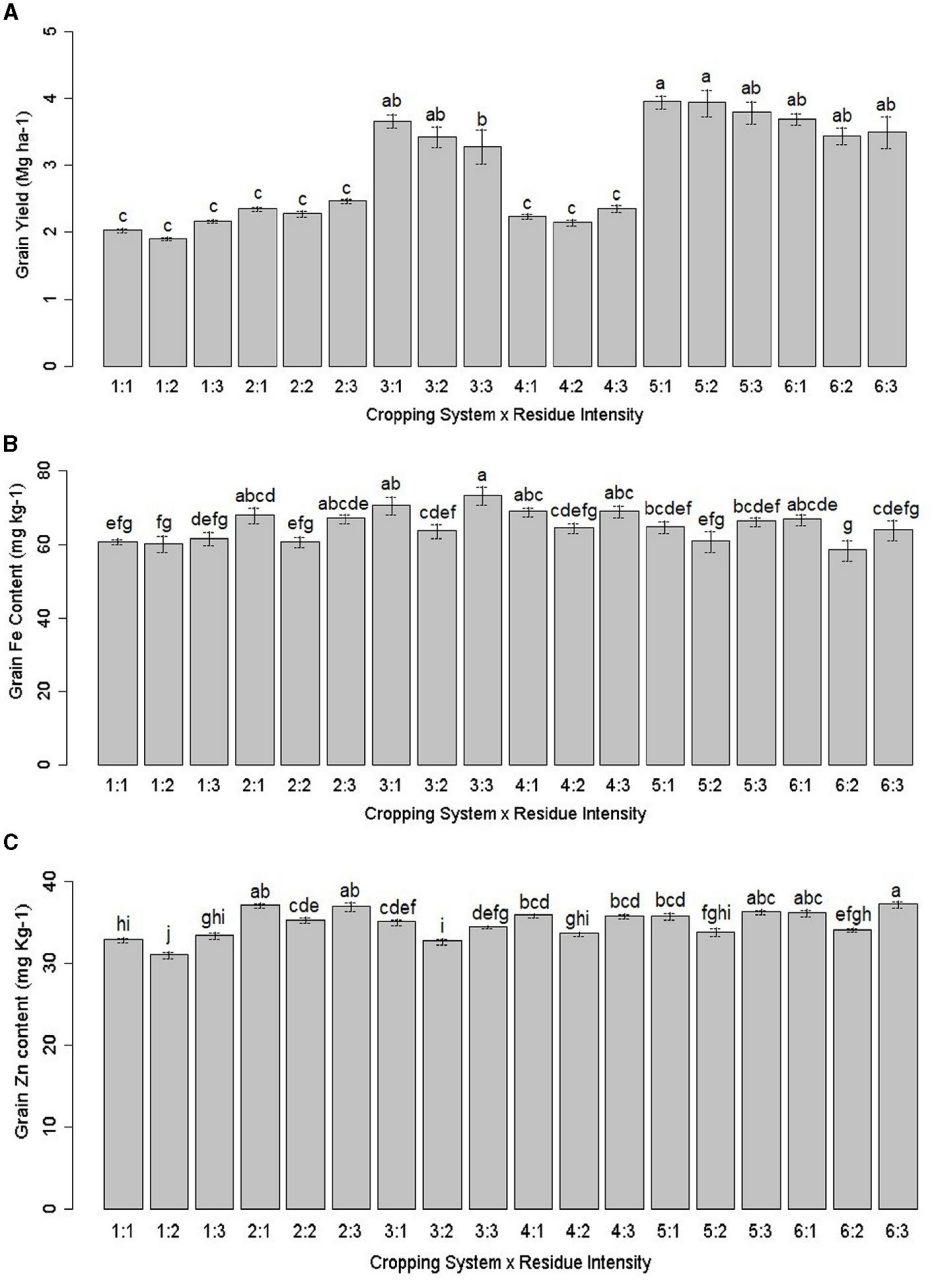
Interactions effect of cropping system and residue intensity on **(A)** pearl millet grain yield (MG ha^−1^), **(B)** grain Fe (mg kg^−1^), and **(C)** Zn content (mg kg^−1^). Treatment details are given in Table 2. Different lowercase letters represent significant differences between different treatments.

The zero-tillage (ZT) system, particularly the ZT pearl millet + cluster bean – mustard cropping system (P+CL–M) and ZT pearl millet + cowpea – mustard + barley cropping system (P + C – M + B), gave the highest productivity (*p* = 0.036) and superior quality of pearl millet compared to other treatments (Tables 3, 4). Treatment ZT-based pearl millet + cluster bean – mustard cropping system [ZT (P + CL – M)] produced the highest pearl millet equivalent yield (PEY) of 3.89 Mg ha^−1^ (pearl millet yield + PEY of intercrop), which was 90.9% higher than the lowest yield of 2.04 Mg ha^−1^ in sole cropped CT-based pearl millet-mustard cropping system [CT (P – M)]. On average, various ZT and system intensification treatments enhanced 49.7% PEY compared to CT (Table 4). In terms of grain yield of pearl millet, the highest yield was obtained with the ZT pearl millet + cowpea – mustard cropping system [ZT (P + C – M)], which was 12.5% higher than CT (P – M), followed by ZT (P + CL – M) > ZT (P – M) (Table 4). The interaction effect analysis highlighted that the ZT-pearl millet – mustard + chickpea cropping system [ZT (P – M + CP)] resulted in the highest grain yield when combined with *Kharif* season crop residue retention (KR), followed by *Rabi* season crop residue retention (RR) practice (Figure 2A). Other cropping systems were statistically at par (Figure 2A and Table 3). ZT (P + C – M) gave a maximum stover yield (7.07 Mg ha^−1^), which was ~10.3% higher than the lowest stover yield of 6.41 Mg ha^−1^ in CT (P – M). On average, the ZT treatments brought ~10.8% improvement in stover yield compared to CT. These results elucidate the significant impact of the interaction between the cropping system and residue intensity on pearl millet grain yield.

**Table 3:** ANOVA for pooled data over year, cropping system (A), residue management (B), and replication (Rep) and their interaction.

**Source**	**Year**	**Rep**	**A**	Year^*^A	A^*^Rep.	**B**	Year^*^B	B^*^Rep	A^*^B	Year^*^A^*^B
DF	1	4	5	5	20	2	2	8	10	10
Grain yield	0.02^**^	0.203^**^	0.244^**^	0.009^**^	0.002	0.439^**^	0	0.002	0.002	0.004^*^
Straw yield	43.61^**^	0.83^**^	1.1^**^	0.01	0.03	1.86^**^	0.13^**^	0.02	0.03	0.02
Grain Zn content	345^**^	9^**^	36^**^	2^**^	0	55^***^	17^**^	0	1	1
Grain Fe content	2097^**^	245^**^	164^**^	7	8	338^**^	24	6	19	39^**^
Grain Mn content	365^***^	18^**^	36^**^	2^*^	1	0	30^**^	2	2^*^	2^*^
Grain Cu content	11^**^	2^***^	11^***^	1^***^	0	4^***^	6^***^	0^*^	0^**^	0
Straw Zn content	3,220^**^	5.3^***^	22.2^**^	2.4^***^	0.4^*^	4.2^**^	7.6^**^	0.3	0.5^*^	0.5^*^
Straw Fe content	6,151.4^**^	532.7^**^	725.6^**^	15.2	10.7	195.2^**^	44.5	5.1	30	43.5^**^
Straw Mn content	421^**^	29.1^**^	36.5^***^	4.8^*^	1.8	12^**^	53.2^***^	1.4	4.5^*^	5.7^**^
Grain Zn uptake	15,404,675^**^	39,510^**^	26,948^**^	18,406^**^	177	39,921^**^	26,152^**^	198	498	484
Grain Fe uptake	12,970^**^	3,489^**^	2,528^*^	52	53	6,596^**^	209^*^	39	98	243^**^
Grain Mn uptake	2,655^**^	950^**^	1,556	65^**^	11	1,496^**^	199^**^	19	11	25^*^
Grain Cu uptake	24^**^	68^**^	161^**^	4^**^	1^**^	198^**^	33^**^	1^**^	2^**^	1
Straw Zn uptake	19,390,512^**^	44,426^**^	89,615^*^	63,330^**^	1,912^*^	82,043^**^	69,358^**^	2,675^**^	1,542^*^	1,606^*^
Straw Fe uptake	20,846^**^	42,536^**^	64,383^**^	501	908	55,309^**^	5,844^**^	94^*^	1,497^**^	2,602^**^
Straw Mn uptake	144,769^**^	8,645^**^	14,172^**^	238	222	15,658^**^	5,129^**^	279	545^*^	500^*^
Straw Cu uptake	21,257^**^	781^**^	1,696^*^	8	47^*^	4,763^**^	6	60^*^	61^**^	81^**^
Oil content (%)	0.03^**^	0	0.08^**^	0.02^**^	0^*^	0.01^**^	0.01	0.02^**^	0.01^**^	0.01^**^
Protein content (%)	0.1	0.09	3.42^**^	0	0.08	0.04	0.39^**^	0.1	0.06	0.12

Values are mean squared (MS). DF: degree of freedom, yield in Mg ha^−1^, nutrient content (mg kg^−1^), nutrient uptake (g ha^−1^); ^*^p = 0.05, ^**^p = 0.05–0.01, ^***^p < 0.01. Cropping system (A), residue management (B), and replication (Rep).

**Table 4:** Effect of cropping systems and residue management on yield and grain quality parameters of pearl millet (2-year pooled data).

**Treatments**	**PEY (Mg ha^−1^)**	**Pearl millet grain yield (Mg ha^−1^)**	**Stover yield (Mg ha^−1^)**	**Grain protein (%)**	**Oil content (%)**	**Oil yield (kg ha^−1^)**	**Protein yield (kg ha^−1^)**
**Cropping system and tillage configuration (main plot)**							
CT (P – M)	2.04^d^	2.04^c^	6.42^d^	10.65^d^	4.97^d^	101.5^d^	217.2^d^
ZT (P – M)	2.37^c^	2.37^a^	7.06^a^	11.00^c^	4.96^d^	117.7^c^	261.2^c^
ZT (P + C – M + B)	3.46^b^	2.20^b^	6.70^c^	11.43^b^	5.05^bc^	174.9^b^	398.5^b^
ZT (P – M + C)	2.25^cd^	2.25^b^	6.83^c^	10.98^c^	4.99^cd^	112.7^cd^	247.7^cd^
ZT (P + CL – M)	3.89^a^	2.25^a^	6.93^a^	11.56^ab^	5.07^b^	197.34^a^	450.3^a^
ZT (P + C – M)	3.54^b^	2.33^a^	7.07^a^	11.82^a^	5.13^a^	182.1^b^	419.3^ab^
**Residue intensity (sub plot)**							
KR	2.99^a^	2.23^b^	6.90^b^	11.22^a^	5.018^a^	150.8^a^	338.7^a^
RR	2.86^b^	2.13^c^	6.58^c^	11.22^a^	5.053^a^	144.8^a^	323.6^b^
BR	2.93^ab^	2.35^a^	7.02^a^	11.28^a^	5.02^a^	147.6^a^	333.3^ab^

Different letters within the same columns are significantly different (Tukey's HSD). Treatment codes are given in Table 2.

### 3.2. Effects of tillage, CRR, and system intensification on grain protein and oil content

The non-significant but highest grain protein content (11.6%) was observed in the ZT (P +CL – M) system, which was 8.1% higher than the lowest content of 10.6% in the CT (P – M) system (Table 4). On average, the CA enhanced the protein content by 5.3% relative to the conventional system of crop establishment. The highest grain protein percentage was observed in the ZT (P + C – M) system among all the treatments, and the difference ranged from 2.2–9.9% compared to the best treatment. Overall, the residue treatments resulted in ~0.7% increase in protein content compared to each other. Therefore, ZT (P + C – M) can be considered the best treatment for achieving high grain protein content. The ZT (P + CL – M) produced the highest protein yield (450.3 kg ha^−1^), whereas the lowest protein yield (217.2 kg ha^−1^) was recorded when the CT (P – M) system was followed. In terms of oil content, a statistically significant difference was observed between CT (P – M) and all other treatments, whereas ZT (P – M + C) and ZT (P + CL – M) were not significantly different from each other (*p* = 0.001). The sub plot treatment of *Rabi* season residue (RR) had the highest oil content at 5.05%, which was 0.7% higher than the lowest content of 5.01% in *Kharif* season residue (KR) (Table 4). Relative to CT, the CA treatments improved the oil content and oil yield by 1.3 and 3.2%, respectively.

### 3.3. Grain and straw micronutrient content and uptake

ZT treatments increased Fe content by 7.2–13.6%, Zn content by 4.9–12.2%, Mn content by 3.1–6.7%, and Cu content by 8.3–16.7% relative to CT (Table 5). The order of grain Fe content was ZT (P + C – M+B) > ZT (P +CL – M) > KR > ZT (P – M + C) > ZT (P – M) > CT (P – M). The Zn content was greater with chickpea integration compared to cluster beans in the PMCS system. Mn content in ZT (P + C – M + B) remained 22.3% higher as compared with CT (P – M) (Table 5). The highest grain contents of Fe, Zn, and Mn were observed in the ZT (P + C – M + B) treatment with KR (Tables 3, 5). Regarding residue intensity, the highest grain contents of Fe, Zn, and Mn were observed in the both season crop residue retention (BR) and KR treatments, while the highest grain contents of Fe and Cu were observed in the ZT (P + C – M + B) and grain Zn and Mn contents in the ZT (P – M) treatment. The grain Fe, Zn, Mn, and Cu uptake was significantly higher in ZT regimes and followed the order of ZT (P + CL – M) > ZT (P + C – M + B) > ZT (P + C – M) > ZT (P – M + C) = ZT (P – M) (Table 5). The interaction effect showed a different trend for grain Fe and Zn content. Fe content was non-significant; however, it was the highest with ZT (P + C – M + B) × BR, followed by ZT (P + C – M) × RR (Figure 2B). As shown in Figure 2C, Zn content was highest in the ZT (P + C– M) × BR combination. Overall, the combination of ZT and KR can enhance the biofortification of pearl millet significantly.

**Table 5:** Effect of cropping system and residue management on micronutrient content (mg kg^−1^) and uptake (g ha^−1^) (2-year pooled data).

**Treatments**	**Grain Fe content (mg kg^−1^)**	**Grain Zn content (mg kg^−1^)**	**Grain Mn content (mg kg^−1^)**	**Grain Cu content (mg kg^−1^)**	**Grain Fe uptake (g ha^−1^)**	**Grain Zn uptake (g ha^−1^)**	**Grain Mn uptake (g ha^−1^)**	**Grain Cu uptake (g ha^−1^)**
**Cropping system and tillage configuration (main plot)**								
CT (P – M)	60.9^d^	32.4^e^	54.0^d^	14.4^d^	124.4^d^	66.3^d^	110.2^d^	29.5^d^
ZT (P – M)	65.3^bc^	36.4^a^	57.6^a^	15.6^c^	155.1^c^	86.4^c^	136.8^c^	37.1^c^
ZT (P + C – M + B)	69.2^a^	34.0^d^	55.7^c^	16.8^a^	239.3^ab^	117.9^b^	192.9^b^	58.3^ab^
ZT (P – M + C)	67.5^ab^	35.0^c^	56.5^bc^	16.1^b^	152.6^c^	79.1^c^	127.5^c^	36.4^c^
ZT (P + CL – M)	64.0^c^	35.2^c^	56.6^b^	15.7^c^	249.6^a^	137.3^a^	220.4^a^	61.4^a^
ZT (P + C – M)	63.1^cd^	35.8^b^	57.8^a^	15.7^c^	224.1^b^	127.1^b^	205.4^ab^	55.8^b^
**Residue intensity (sub plot)**								
KR	66.7^a^	35.4^a^	56.4^a^	15.9^a^	200.4^a^	106.4^a^	169.2^a^	48.0^a^
RR	61.5^b^	33.4^b^	56.2^a^	15.4^b^	175.6^b^	95.8^b^	161.5^b^	44.3^b^
BR	66.9^a^	35.6^a^	56.4^a^	15.9^a^	196.6^a^	104.9^a^	165.9^ab^	46.9^a^

Different letters within same columns are significantly different (Tukey's HSD). Treatment codes are given in Table 2.

### 3.4. GGE biplot and stability analysis

#### 3.4.1. Genotype × trait biplot and genotype × yield × trait biplot

**Figure 3:**
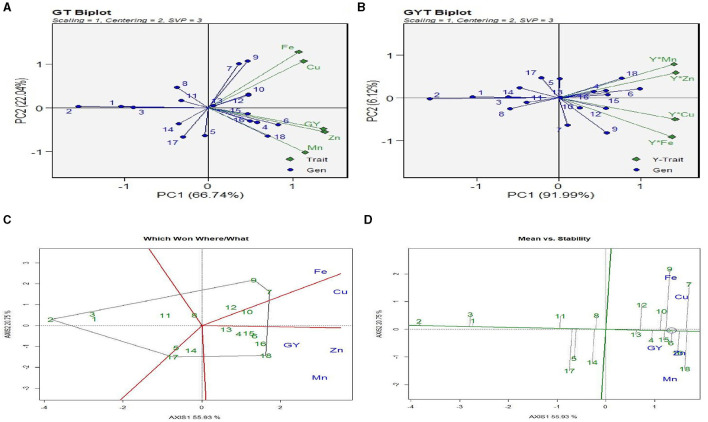
GGE analysis of interaction between treatments, micronutrients content, and grain yield. **(A)** GT plot: treatments × grain yield × micronutrient content (Fe, Zn, Cu, and Mn). **(B)** GYT biplot: treatments × (grain yield × micronutrient). **(C)** Which Won Where/What polygon. **(D)** Mean vs. stability biplot. Treatment details are given in Table 2.

The “genotype (here treatment) × trait (GT)” biplot (Figure 3A) displays the association between various traits. The biplot exhibited high accuracy, having 88.8% goodness of fit. The angle among the trait vectors was <90°, indicating a positive correlation between all the traits. The biplot shows that treatments 18 and 13 (see Table 2 for treatment details) had high Fe and Cu content. The grain Fe and Cu content was the lowest with treatments 11 and 12. Grain yield contents of Zn and Mn were the highest with treatments 18 and 14 and the lowest with treatments 9 and 10. The genotype × yield (Y) × trait (GYT) biplot (Figure 3B) was used to select the treatments on the basis of their relative performance. The goodness-of-fit for the GYT biplot for grain yield and nutrient content was 98.1%. Treatment 18 and 14 combinations had the most significant values for Y × Mn and Y × Zn, indicating that these treatments were best combined for grain yield with Mn and Zn content. Similarly, treatment 18 had the highest levels of Y × Fe and Y × Cu, meaning that this treatment was the best combination for grain yield with Fe and Cu content.

#### 3.4.2. Which-Won-Where/What polygon

**Figure 4:**
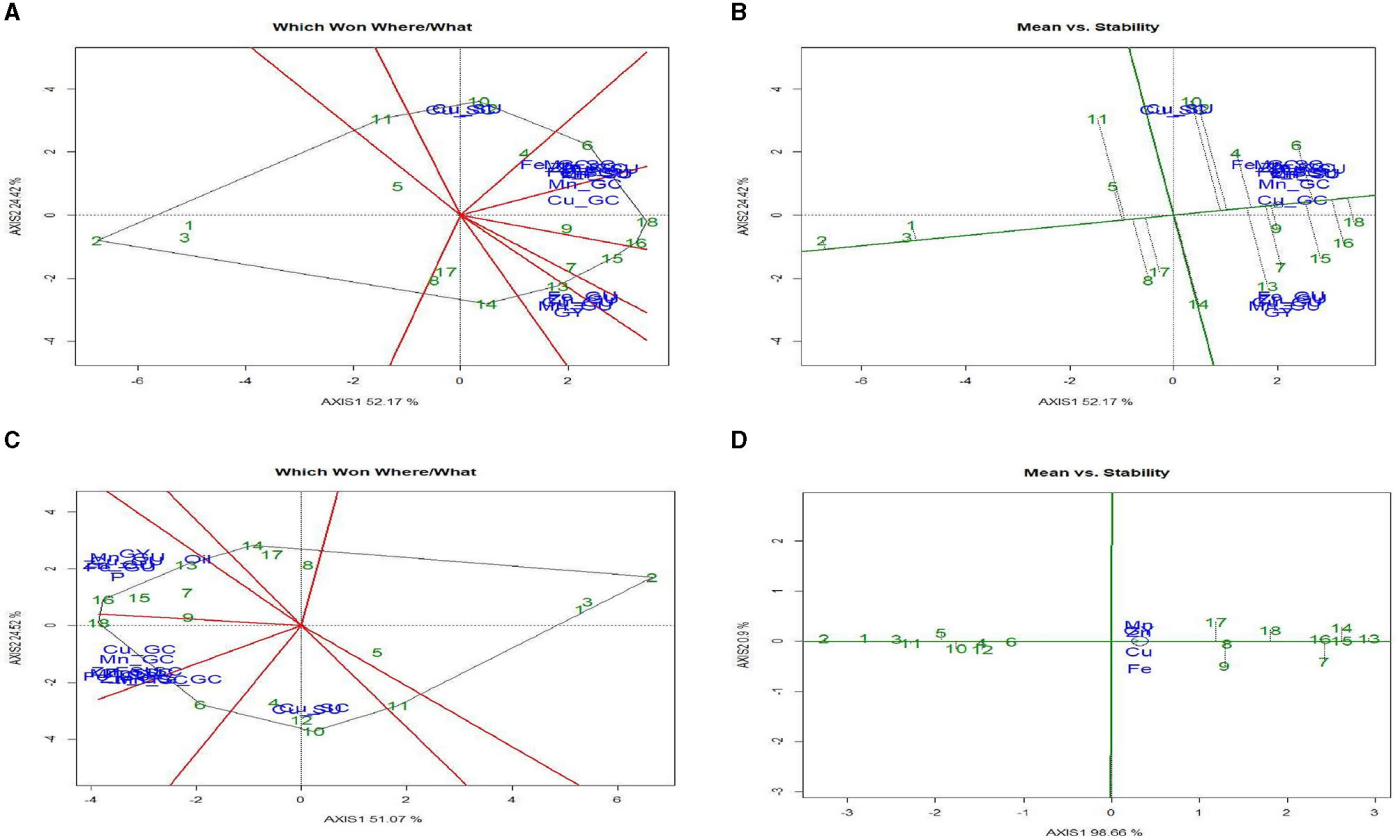
GGE analysis for grain yield (GY) and micronutrients content and uptake (Fe, Zn, Cu, and Mn). **(A)** Which Won Where/What polygon. **(B)** Mean vs. stability biplot; GGE analysis for grain, yield, straw yield, micronutrient content and uptake, oil and protein content. **(C)** Which Won Where/What polygon. **(D)** Mean vs. stability biplot for micronutrient grain uptake. Treatment details are given in Table 2.

The “Which-Won-Where/What” polygon (Figure 3C) was used to find out the best treatments with respect to grain yield and micronutrient content (NC). The first two principal components (PC1 and PC2) explained 76.8% variation between treatment and trait. The biplot had five different sectors, but all the traits were located only in two sectors. Fe and Cu were located in the same sector, and treatment 9 [ZT (P + C – M + B)^*^RR] was the best treatment (farthest treatment) for these nutrients, followed by treatment 7. Grain yield (GY), Zn, and Mn content were placed in another segment, and treatment 18 [ZT (P + C – M)^*^BR] was the vertex treatment in the segment, indicating that this treatment performed best for these three traits. Treatment 2 [ZT (P – M) × KR] was the poorest treatment for all the traits. The “Which-Won-Where/What” polygon of Figure 4A shows the best treatments for grain yield and micronutrient uptake (NU) in grain and stover. The first two principal components (PC1 and PC2) explained a 75.6% variation in treatment × trait. All the traits were located only in four segments. Fe, Cu, and Mn uptake in grain and the GY were consolidated, and treatments 13, 14, 15, and 16 were the best for these traits, followed by treatment 7. Fe, Mn, and Cu uptake in grain and stover was strongly correlated and situated in one sector, and treatments 18 and 6 were outperformers. The polygon in Figure 4C shows the pooled result of quality aspects of nutrient content and uptake, oil, and protein content with grain yield, eliminating stover yield and stover uptake traits. Out of the eight sectors, all traits were observed in four sectors only. Grain yield, protein content, and uptake of Fe, Zn, Mn, and Cu in the grain were correlated, and the best treatment for this pooled trait was treatment 16, followed by treatments 15 and 7. Oil content (%) was observed in a decent number of other traits, and the best treatment was treatment 13 [CT (P – M) × BR].

#### 3.4.3. Mean vs. stability biplot

The “Mean vs. Stability” biplot (Figure 3D) was used to identify the highly stable treatment among all the traits under study. The treatment stability was inversely related to the magnitude of the projection on the typical environment coordinate. Across the traits, treatments 1, 2, and 3 were highly stable, but their performance was poor. Treatments 7, 9, and 18 had the highest values of the traits but were highly unstable in their performance, whereas treatment 10 had a higher content of Fe and Cu, which was also highly stable. Likewise, treatment 16 was relatively stable and had higher GY, Zn, and Mn values. The “Mean vs. Stability” biplot (Figure 4B) was used to determine the highly stable GY, NC, and NU treatments. Across the treatments, 1, 2, and 3 were highly stable but poor performers. Treatments 14, 15, 6, and 4 had the highest GY, NC, and NU pooled grain and stover values. However, treatments 9, 18, and 16 were highly stable for higher GY and grain NC. The “Mean vs. Stability” biplot (Figure 4D) was also used to determine the stability between grain yield and uptake of Zn, Fe, Mn, and Cu. Treatments 8, 10, 13, and 16 were highly stable but performed poorly in terms of grain yield and micronutrient uptake, whereas treatments 7, 14, 1, and 18 showed better performance and stability.

### 3.5. Grain fatty acid composition

**Figure 5:**
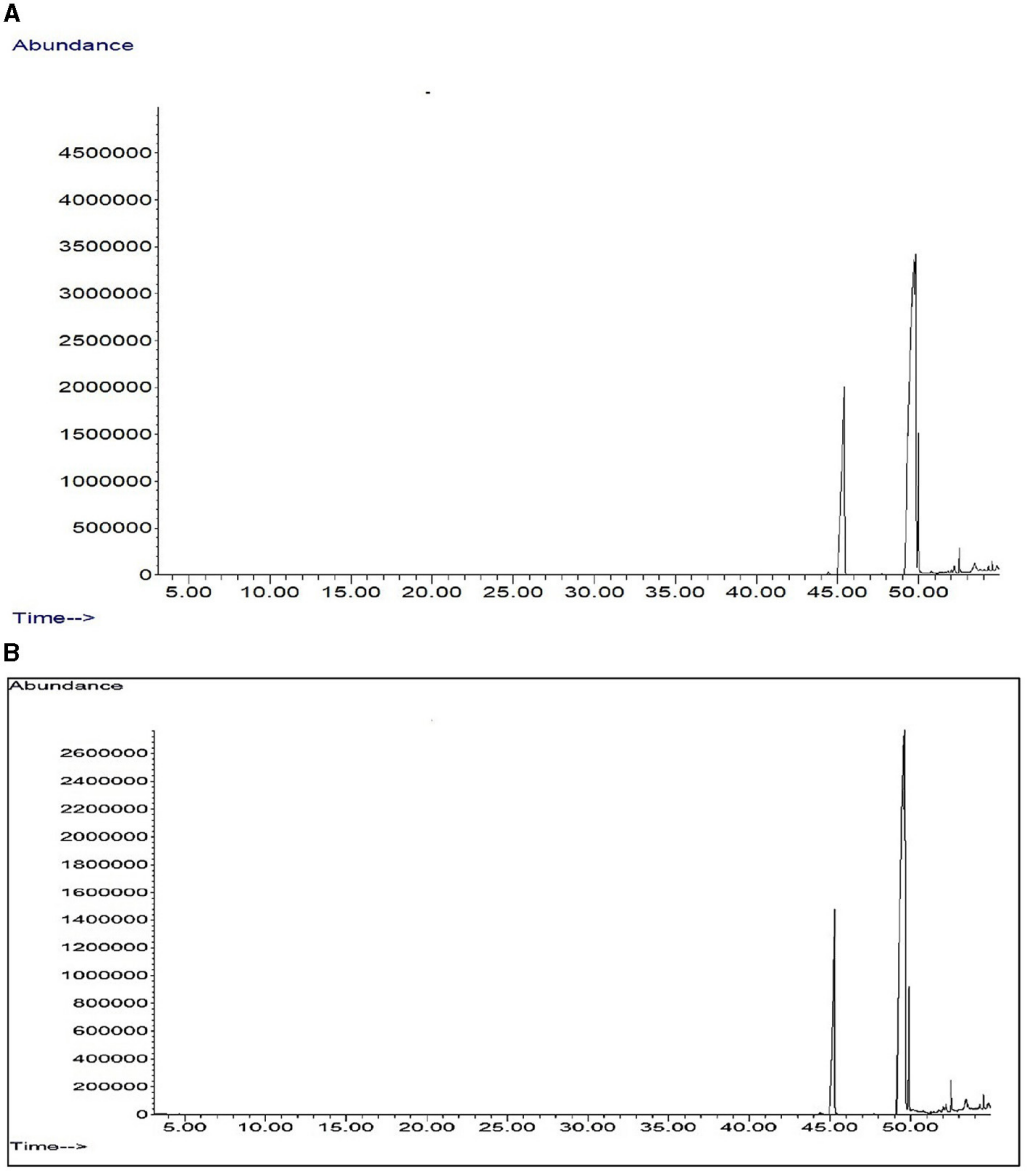
GC-MS total ion chromotogram of fatty acid profiling of pearlmillet oil in **(A)** zero tillage regimes and **(B)** conventional tillage system.

The fatty acid composition analysis of pearl millet showed significant differences between ZT with crop residue retention and CT with incorporated crop residue. The most notable difference was observed in the percentage content of (9Z, 12Z) 9,12-octadecadienoic acid (linolic acid), which increased from 55.9% in CT to 58.7% in ZT with crop residue retention (Table 6). This indicated that ZT with crop residue retention positively impacts quality composition by promoting the accumulation of this unsaturated fatty acid, which is known to play a crucial role in maintaining human health. Furthermore, the increase in 9,12-octadecadienoic acid was accompanied by a decrease in the percentage content of 10-octadecenoic acid, which decreased from 25.0% in CT to 16.8% in ZT (Figures 5A, B) with crop residue retention.

**Table 6:** Effect of tillage and cropping systems on management on fatty acid profile of pearl millet crop (2-year pooled data).

**S.N**.	**Retention time**	**Compound**	**Relative percentage (%)**
**Zero tillage with soil surface retention of crop residues**			
01	45.42	Hexadecanoic acid	21.0
02	49.71	9,12-Octadecadienoic acid	58.7
03	49.82	9-Octadecenoic acid	16.8
04	49.99	Octadecanoic acid	3.5
**Conventional tillage with crop residue-incorporation**			
01	45.29	Hexadecanoic acid	19.1
02	49.54	9,12-Octadecadienoic acid	56.0
03	49.66	10-Octadecenoic acid	25.0

## 4. Discussion

### 4.1. Effect on crop productivity and grain quality

Crop production performance is influenced by various factors such as soil water, fertilizer availability, gas exchange, heat supply, and ultimately the economic yield of the crop (46–48). In rainfed conditions, enhancing crop productivity requires the conservation of soil moisture, increased nutrient availability, optimal soil health, and efficient utilization of solar radiation. The evaluation of long-term tillage and diverse cropping systems allows for a comprehensive assessment of the advantages and disadvantages of tillage practices and system intensification effects while also considering crop cycles and yield stability on a broader scale (48, 49). The findings of this study demonstrate that adopting ZT and residue retention techniques has a positive impact on pearl millet equivalent yield (PEY) and stover yield. These practices contribute to increased moisture and nutrient availability, as well as improved infiltration due to the retention of crop residues (7, 50). These results align with previous studies that have shown the stabilizing effect of ZT with crop residue retention on yield and grain quality of pearl millet crops (9, 13). Furthermore, the implementation of ZT and residue retention promotes higher microbial activity and diversity, leading to improved soil nutrient availability. Rational system intensification and appropriate tillage practices also contribute to improved soil quality by fostering better soil structure and reducing soil bulk density (51, 52). Crop residue retention plays a crucial role in building soil organic matter (SOM) and reducing mineral-N losses (15, 53, 54). In comparison to CT, conservation tillage practices like ZT with residue mulching hold promise as they provide favorable conditions for moisture, nutrient availability, and soil structure, thereby ensuring high and stable crop yields (46).

The quality of pearl millet grain was assessed in terms of oil and protein content. The highest grain quality was observed in systems combining ZT with legume intensification, such as the incorporation of cowpea or cluster bean. Legumes are well-known for their ability to fix atmospheric nitrogen through root nodules and increase microbial activity in the rhizosphere. Legumes also contribute to soil organic matter through leaf fall, ultimately enhancing soil chemical and microbial health and thereby promoting increased yield and grain quality (45, 55). Higher protein content in grain is often associated with increased nitrogen availability to the plant, a correlation that was also observed in this experiment (56, 57). The ZT and residue retention systems exhibited higher levels of available sulfur (S) compared to the conventional tillage system. The build-up of soil organic carbon (SOC) and available sulfur contributed to a higher oil content (58). The improved oil concentrations, coupled with a higher grain yield, resulted in a higher oil yield. Overall, the combination of ZT, residue retention, and legume inclusion proved to be a beneficial nexus, leading to improved crop yield. The proposed study holds merit in addressing key knowledge gaps, providing practical insights for improving agricultural practices, and enhancing the nutritional value and resilience of pearl millet production in rainfed agro-ecosystems.

### 4.2. Micronutrient content and uptake

This study investigated the effect of ZT, residue retention, and system intensification on micronutrient content and uptake in the soil-plant system. Micronutrients in the soil are often present in forms that are not readily available to plants, particularly in their native cationic form. However, the combination of ZT and crop residue retention (CRR) practices in this study facilitated improved availability and uptake of certain micronutrients, such as iron (Fe) and manganese (Mn), which exist in reduced forms (Fe^2+^ and Mn^2+^). The increased soil moisture under CRR on the soil surface favored the reduced forms of these nutrients and facilitated their movement and diffusion from the soil to plant roots (59). Moreover, the better moisture conservation achieved under ZT and CRR promoted the uptake of micronutrients that move via mass flow into the plant system and subsequently translocate to the grain. In contrast, CT practices led to the mineralization of soil organic carbon (SOC) and limited the complexation of micronutrients with SOC, resulting in lower micronutrient concentrations.

Several previous studies have reported higher concentrations of Zn, Cu, Fe, and Mn under the ZT system with residue retention (14, 60, 61). Consistent with these findings, our study also observed higher grain nutrient content and grain yield, leading to significantly higher micronutrient uptake. A similar pattern was reported in a study focused on a no-till-based lentil cropping system (53). Additionally, micronutrient build-up in the soil under ZT and CRR on the soil surface has been reported, indicating replenishment of the micronutrient pool in real time (62, 63). The combination of ZT, CRR, and intercropping practices increases the availability of micronutrients, including Zn, Fe, Mn, and Cu, in both extractable and organic forms (14, 61). The improved microbial activity observed in CRR plots with legume intercropping likely contributed to the increased solubilization of micronutrient cations (53). The formation of organic complexes between micronutrient cations and organic acids generated during the decomposition of plant residues is believed to be responsible for the increased micronutrient content, particularly Zn and Cu, in plots with residue retention (9, 64, 65). In CT practices, crop residues are often not recycled, resulting in reduced carbon (C) input and the loss and depletion of nutrients in the soil. In contrast, CA practices disturb the soil less, and the associated crop residue retention in this system leads to nutrient recycling and increased C input. These practices also stimulate microbial and enzymatic activity in the soil.

Legume-based systems, due to nitrogen fixation and faster biomass decomposition with a narrow carbon-to-nitrogen (C:N) ratio, increase carbon sequestration and enhance micronutrient acquisition (49). The resulting soil organic matter (SOM) may also have facilitated the synthesis of organic acids in the rhizosphere, which acted as micronutrient chelates, influencing the translocation and remobilization of micronutrients (66).

### 4.3. Fatty acid composition

Generally, fatty acids give a unique flavor to food. Pearl millet is known to contain higher levels of fatty acids compared to other cereal grains. Linoleic and oleic acids are two of the essential fatty acids present in pearl millet. The balanced compositions of these fatty acids are essential for the oil's stability and the quality of the grain (67). The impact of farm management practices on fatty acid levels in grain needs to be better understood. In this study, we observed differences in fatty acid content between different tillage practices. Drought stress has been reported to increase the oleic acid and decrease the linolic acid content (68, 69). Hexadecanoic acid possesses antioxidants, anti-inflammatory, and hypocholesterolemic properties. Di-linoleic acid (9,12-octadecadienoic acid) has been reported to have anti-arrhythmic properties (67, 68). These findings suggest that ZT with crop residue retention can help improve soil quality and health impacts in diets by altering the fatty acid composition, which can significantly impact the nutritional value and productivity of pearl millet crops. This aligns with our findings because ZT coupled with CRR increased the moisture availability and thus decreased the oleic acid and linolic acid content in pearl millet grain. However, further studies are required to understand the role of farm practices on fatty acid composition.

## 5. Conclusion

The combination of residue retention in the zero-tillage system along with system intensification using leguminous crops shows great potential for enhancing micronutrient biofortification and achieving stable yields in pearl millet-based cropping systems. The success of this sustainable approach relies on effective soil moisture conservation, improved soil chemical and biological health, and increased system productivity. The interactive effects of tillage and residue recycling play a significant role in micronutrient biofortification, while yield stability is primarily influenced by tillage and system intensification practices. This sustainable approach offers a promising solution to address the challenges of micronutrient malnutrition and low crop yields in rainfed dryland areas. For future research, it is essential to gain a deeper understanding of the hydro-thermal dynamics under zero tillage and the impact of residue management in diverse intercropping systems on nutrient distribution in different plant parts. Additionally, investigating the effects of residue mulching and legume intercropping on greenhouse gas emissions from all crops within each system will provide valuable insights for advancing sustainable agricultural practices.

## Data availability statement

The original contributions presented in the study are included in the article/Supplementary material, further inquiries can be directed to the corresponding authors.

## Author contributions

AYo: conceptualization, methodology, investigation, monitoring, data curation, and writing of the original and final draft. RB: conceptualization, investigation, review, writing, and editing. SG: review and editing and data analysis. RN: draft finalization and editing. SS: data curation. AC: methodology conceptualization, investigation, review, writing, and editing. YS: review and methodology. DS: data analysis and data curation. SB: writing and review. TS: writing and editing. AYa: draft initiation and supervision. SN: methodology and analysis. NS: investigation, review, and editing. All authors contributed to the article and approved the submitted version.

## Footnotes

^1^WHO (2022). https://www.who.int/news/item/06-07-2022-un-report–global-hunger-numbers-rose-to-as-many-as-828million-in-2021 (accessed June 28, 2023).
