# Supplementary material for: Elucidating the interactive impact of tillage, residue retention and system intensification on pearl millet yield stability and biofortification under rainfed agro-ecosystems
Source: Front Nutr. 2023 Aug 21;10:1205926. doi: 10.3389/fnut.2023.1205926 (PMC10475997; doi:10.3389/fnut.2023.1205926)
Supplement: Supplementary file 1 [file Data_Sheet_1.docx]

**Tillage, residue-recycling and system-intensification nexus under rainfed agro-ecosystem: implications on pearlmillet yield stability and biofortification**

**Akshay K. Yogi**†**^1^, Ram Swaroop Bana**†**^1*^, Samarth Godara^2^, Seema Sangwan^1^, Anil K. Choudhary^1,3*^, Ravi C. Nirmal^1^, Shanti D. Bamboriya^1,4^, Y.S. Shivay^1^, Teekam Singh^1^, Achchhelal Yadav^1^, Shivani Nagar^1^, Nirupma Singh^1^**

| Replication | Replication 1  **6 m**  **6 m**  **6 m** | | |  | Replication 2 | | |  | Replication 3 | | |
| --- | --- | --- | --- | --- | --- | --- | --- | --- | --- | --- | --- |
| Residue Management | KR@30% | RR@30% | BR@30% |  | KR@30% | RR@30% | BR@30% |  | KR@30% | RR@30% | BR@30% |
| Cropping system | CT (P + M) | | |  | CT (P + M) | | |  | CT (P + M) | | |
|  | ZT (P + M) | | |  | ZT (P + M) | | |  | ZT (P + M) | | |
|  | ZT (P + C – M + B) | | |  | ZT (P + C – M + B) | | |  | ZT (P + C – M + B) | | |
|  | ZT (P + M+ CH) | | |  | ZT (P + M+ CH) | | |  | ZT (P + M+ CH) | | |
|  | ZT (P + CL – M) | | |  | ZT (P + CL – M) | | |  | ZT (P + CL – M) | | |
|  | ZT (P+C – M) | | |  | ZT (P+C – M) | | |  | ZT (P+C – M) | | |

^1^ICAR-Indian Agricultural Research Institute, New Delhi India

^2^ICAR-Indian Agricultural Statistics Research Institute, New Delhi India

^3^ICAR-Central Potato Research Institute Shimla, Himachal Pradesh, India

^4^ICAR-Indian Maize Research Institute, Ludhiana, Punjab, India

**Supplementary Figure 1**. **Layout plan of field experiment.**

**24 m**

**24 m**

**24 m**

**Supplementary Figure** 2. Sowing, harvesting and crop period timeline for the experiment.

**Supplementary Table 1.** Nutrient composition (dry weight basis) of crop residues

| Residue | Applied season |  | Nutrient content (%) | |  |
| --- | --- | --- | --- | --- | --- |
|  |  | N | P | K | S |
| Mustard | *Kharif*, 2020 | 0.47 | 0.14 | 1.07 | 0.25 |
|  | *Kharif*, 2021 | 0.51 | 0.16 | 1.12 | 0.31 |
| Pearl millet | *Rabi*, 2020-21 | 0.64 | 0.18 | 1.31 | 0.14 |
|  | *Rabi*, 2021-22 | 0.69 | 0.17 | 1.33 | 0.16 |

**Supplementary Table 2.** Minimum support price of the crops

| Crop | Price ($/Kg) grain yield |
| --- | --- |
| Pearl millet | 0.30 |
| Cowpea | 0.59 |
| Clusterbean | 0.55 |
